# Overexpression of miR-24 Is Involved in the Formation of Hypocoagulation State after Severe Trauma by Inhibiting the Synthesis of Coagulation Factor X

**DOI:** 10.1155/2017/3649693

**Published:** 2017-06-14

**Authors:** Lu-Jia Chen, Lian Yang, Xing Cheng, Yin-Kai Xue, Li-Bo Chen

**Affiliations:** ^1^Department of Emergency Surgery, Union Hospital, Tongji Medical College, Huazhong University of Science and Technology, Wuhan 430022, China; ^2^Department of Radiology, Union Hospital, Tongji Medical College, Huazhong University of Science and Technology, Wuhan 430022, China

## Abstract

**Background:**

Dysregulation of microRNAs may contribute to the progression of trauma-induced coagulopathy (TIC). We aimed to explore the biological function that miRNA-24-3p (miR-24) might have in coagulation factor deficiency after major trauma and TIC.

**Methods:**

15 healthy volunteers and 36 severe trauma patients (Injury Severity Score ≥ 16 were enrolled. TIC was determined as the initial international normalized ratio >1.5. The miR-24 expression and concentrations of factor X (FX) and factor XII in plasma were measured. In vitro study was conducted on L02 cell line.

**Results:**

The plasma miR-24 expression was significantly elevated by 3.17-fold (*P* = 0.043) in major trauma patients and reduced after 3 days (*P* < 0.01). The expression level was significantly higher in TIC than in non-TIC patients (*P* = 0.040). Multivariate analysis showed that the higher miR-24 expression was associated with TIC. The plasma concentration of FX in TIC patients was significantly lower than in the non-TIC ones (*P* = 0.030) and controls (*P* < 0.01). A negative correlation was observed between miR-24 and FX. miR-24 transduction significantly reduced the FX level in the supernatant of L02 cells (*P* = 0.030).

**Conclusions:**

miR-24 was overexpressed in major trauma and TIC patients. The negative correlation of miR-24 with FX suggested the possibility that miR-24 might inhibit the synthesis of FX during TIC.

## 1. Introduction

Traumatic injury is life threatening and it is the fourth leading cause of death in high-income countries [1, 2]. Trauma-induced coagulopathy (TIC) is recognized as an early hypocoagulable state leading to exacerbation of bleeding and associated with fourfold-increased mortality among severely injured patients [3]. TIC was first described during the Korean War [4]. Its complex fundamental mechanisms remain elusive up till now [5]. Current opinions suggest that the pathogenesis is multifactorial and mechanisms like activation of the protein C pathway, endothelial injury, coagulation factor deficiency, hyperfibrinolysis, and platelet dysfunction participate in the development of TIC [6–8].

Coagulation factor deficits after severe trauma have been recognized by a number of studies for many decades [9–13]. It is verified by the successful therapies of repletion of clotting factors by infusion of higher ratio of flesh frozen plasma (FFP) and additional prothrombin complex concentrate (PCC) in trauma patients [14–16]. Studies have shown that coagulation factor deficiency occurred early on the scene and aggravated with time [12, 17]. Theories explaining this phenomenon are direct loss, ongoing consumption, and dilution [6, 18]. However, reduction in the synthesis of coagulation factor can also be present.

MicroRNAs (miRNAs) are noncoding small RNA molecules that are 20–23 nucleotides in length, which play important regulatory roles in plants and animals by targeting mRNAs for cleavage or translational repression. They regulate diverse cellular functions including proliferation, differentiation, apoptosis, and plasticity [19]. miRNAs have lately been recognized to play a role in severe injuries. Yet, few reports have expounded on that subject [20, 21]. A recent study implied that miRNA-24-3p (miR-24) can reduce the FX and FXII mRNA levels by downregulating protein hepatocyte nuclear factor 4*α* (HNF-4*α*) [22]. The clinical relevance of this particular finding is however still not clear. So in the present study, we investigated the relations between plasma miR-24 expression and coagulation factor X (FX) and XII (FXII) levels in major trauma and TIC patients. We assumed that the overexpression of miR-24 in trauma patients is involved in the hypocoagulation state by inhibiting the synthesis of FX and/or FXII.

## 2. Methods

### 2.1. Patients and Normal Donors

Upon approval from the ethics committee and obtaining written informed consents, blood samples were obtained from 36 severely injured patients from June to August, 2016 at Wuhan Union Hospital, a tertiary hospital located in the center of China. The patients were only included when the researchers were on shifts. All patients enrolled had an Injury Severity Score (ISS) of at least 16 points within 12 h of injury and without any previous medical history. Exclusion criteria were patients aged less than 16 years old, pregnant women, or those who were on anticoagulant medications or had surgeries prior to attending the emergency department (ED). 15 healthy adult donors served as the control group. The patients' standard blood test values, ISS, and shock index (SI) were collected and calculated based on clinical records upon their ED arrivals. TIC was defined by international normalized ratio (INR) of more than 1.5 [23, 24]. The ISS calculation was based on the ordinal scale developed by Baker et al. [25] and updated in 2008.

### 2.2. Blood Collection and Measurements

Blood samples were collected from each patient via venous puncture into tubes containing EDTA at 2 time points: upon arrival to ED and 3 days later if they were still hospitalized. All blood samples were processed for the isolation of plasma within 4 h after collection. Plasma samples was transferred to an RNase-free tube and stored at −80°C. Samples were analyzed by researchers who were blinded to all patients' data.

### 2.3. RNA Isolation and Real-Time Quantitative Reverse Transcription-Polymerase Chain Reaction (qRT-PCR)

For measuring miR-24, total RNA was isolated from 200 *μ*L plasma samples using the RNAmisi microRNA fast extraction kit (Aidlab, Beijing, China) according to the manufacturer's instructions. A synthetic miRNA cel-miR-39 (Biomics, Nantong, China) was added to each plasma specimen at a final concentration of 1 *μ*M as a reference before isolation [26]. Relative quantification of the total RNA was conducted with a 2-step method: reverse transcription (RT) and qRT-PCR. RT reaction was performed using a RevertAid First Strand cDNA Synthesis Kit (Invitrogen, Shanghai, China). 4 *μ*L of extracted RNA, 1 *μ*L, 2 *μ*mol/L stemloop RT primer (2 *μ*M; Invitrogen, Shanghai, China), and 1 *μ*L RNase-free H_2_O, were incubated at 65°C for 10 min. Next, 5× buffer (2 *μ*L), 10 mM dNTP (0.5 *μ*L), RiboLock RNase Inhibitor (0.5 *μ*L), and RevertAid Reverse Transcriptase (1 *μ*L; Thermo Fisher Scientific Inc., Shanghai, China) were added. The samples were incubated at 42°C for 1 h, followed by 10 min at 70°C for enzyme inactivation, after which the reactions were held at 4°C until use. qRT-PCRs were processed in 96-well plates on an ABI StepOnePlus analyzer (Applied Biosystems, CA, USA). In a final reaction volume of 20 *μ*L, the following were added: 0.5 *μ*L of cDNA, 10 *μ*L of qPCR Master Mix (Invitrogen, Shanghai, China), 1.6 *μ*L of miRNA-specific forward and reverse primer (10 *μ*M), and 7.9 *μ*L of nuclease-free water. The reactions were incubated at 95°C for 10 min, followed by 40 cycles of 95°C for 15 s, 60°C for 30 s, and 72°C for 30 s. Each qRT-PCR was performed in triplicate.  Table 1 summarizes the sequences of RT and PCR primer used. Expression values were normalized using the mean threshold cycle (Ct) obtained from the spiked-in controls cel-miR-39. Delta Ct is the difference in the cycling threshold between miR-24 and cel-miR-39. In this article, the relative expression value of miR-24 is either expressed as log_10_2^−Delta Ct^ [27] or −Delta Ct.

### 2.4. Enzyme-Linked Immunosorbent Assay (ELISA)

ELISA was used to detect the plasma levels of FX and FXII; the kits were purchased from Abcam, USA. All the plasma sample processing, measurement, and content calculation were done according to kit instructions.

### 2.5. Cell Culture and Transfections

L02 cells (China Cell Culture Center, Shanghai, China) were cultured in RPMI1640 medium at 37°C under 5% CO_2_. Medium was supplemented with 10% fetal calf plasma, 100 *μ*g/mL streptomycin, and 100 U/mL penicillin. The miR-24 mimics and negative control (NC) sequences were obtained from RIBOBIO (Guangzhou, China). The cells were seeded at a density of 80,000 cells/well one day before transfection using a Lipofectamine 2000 Reagent (Invitrogen, Shanghai, China) according to the manufacturer's protocol. The final concentration of miR-24 mimics was 100 nM. Cell supernatants were harvested at 24 h after transfection. Each group contains 5 examples.

### 2.6. Statistical Analyses

Statistical analysis was performed by SPSS 22 software and *P* values of <0.05 were defined as statistically significant. Fisher's exact test was used for comparisons of proportions. Kolmogorov-Smirnov test was conducted to determine whether the data were normally distributed. For normally distributed variables, data were expressed as mean and standard deviation (SD); the comparisons of 2 groups were analyzed by Student's *t*-test, and 3 groups or more by one-way ANOVA test; the correlation study was conducted by using Pearson's correlation analysis. For the data with a non-normal distribution, data were expressed as mean and range; the comparison of 2 independent groups was analyzed by nonparametric Mann-Whitney *U* test. Binary multivariate logistic regression analysis was used to identify the association of TIC with ISS, SI, and miR-24 level. Repeated measures ANOVA was used to compare the changes of relative values of miR-24 over time. All experiments in the study were performed in duplicate.

## 3. Results

### 3.1. Demographic Data

The present study included 36 trauma patients with the average age of 47.6 ± 13.0 years, of whom 72.22% (*n* = 26) were men. ISS was 29.6 ± 10.7; 80.56% (*n* = 29) of the patients had blunt trauma. Among the 36 patients, 8 patients (22.22%) had TIC, and 9 patients (25%) received massive transfusion (≥10 RBC during the initial 24 hours). Overall mortality was 11.11% (*n* = 4). 2 deaths were due to traumatic brain injury (3 h and 4 d after ED visit), 1 exsanguination (5 h after ED visit), and 1 sepsis syndrome (29 d after ED visit). 15 healthy volunteers were enrolled and with the average age of 42.27 ± 14.59 years; 73.33% were male. Coagulopathic patients have higher ISS, SI, transfusion rate, and platelet requirement as compared to noncoagulopathic patients (Table 2).

### 3.2. Overexpression of Plasma miR-24 Is Found in Major Trauma and TIC Patients

**Figure 1:**
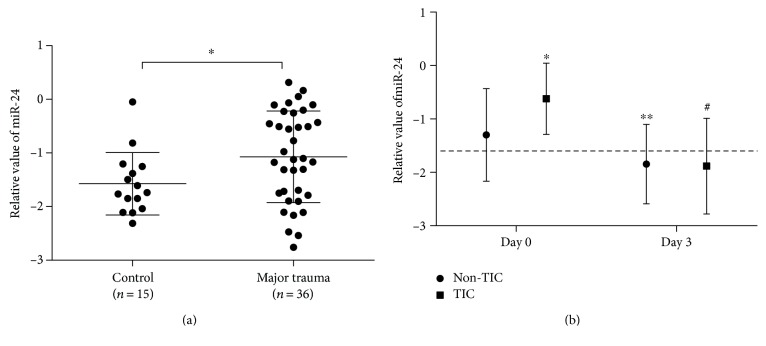
(a) Comparison of plasma expression levels of miR-24 in 36 major trauma patients and 15 controls. Plasma expression levels of miR-24 were higher in all major trauma patients than in healthy controls. ^∗^*P* < 0.05. (b) Plasma expression levels of miR-24 were significantly increased in 8 TIC patients than in the 28 non-TIC ones on ED arrival. MiR-24 expression levels were significantly downregulated on day 3 compared with those on day 0 (*n* = 24 in the non-TIC day-3 group and *n* = 7 TIC day 3 group). Compared to the non-TIC day 0 group, ^∗^*P* < 0.05, ^∗∗^*P* < 0.01, compared to the TIC day 0 group, ^#^*P* < 0.01.

Using qRT-PCR, plasma miR-24 levels were analyzed in the patients and controls. It was identified that average plasma miR-24 expression exhibited a 3.17-fold increase (*P* = 0.043) in all major trauma patients (Figure 1(a)). Plasma expression of miR-24 was significantly higher in TIC patients than in the non-TIC ones (*P* = 0.040). There were 1 death and 3 discharges within 3 days after ED visits in the non-TIC group and 1 death in the TIC group, so that left *n* = 24 and 7 in the two day 3 groups. MiR-24 expression was obviously downregulated on day 3 compared with that on day 0 (*P* < 0.01; Figure 1(b)). In multivariate logistic analysis, greater ISS and higher miR-24 expression were associated with TIC (Table 3).

### 3.3. Concentrations of FX and FXII Were Markedly Reduced in TIC Patients

**Figure 2:**
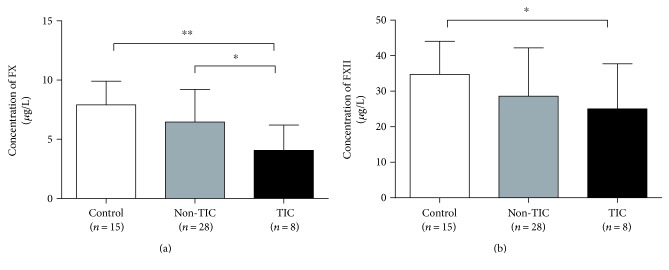
(a) The average level of FX in 8 TIC patients was significantly lower than that in 15 controls and 28 non-TIC patients. (b) Reduction in the average level of FXII in TIC patients compared to that in healthy controls was also noted. ^∗^*P* < 0.05, ^∗∗^*P* < 0.01.

Major trauma patients were reported to have coagulation factor deficiency. As shown in Figure 2(a), the average level of FX was significantly reduced by 48.63% in TIC patients than in healthy controls (4.06 ± 2.14 versus 7.91 ± 2.00 *μ*g/L, *P* < 0.01). The FX level was lower in TIC patients than in the non-TIC ones (4.06 ± 2.14 versus 6.46 ± 2.76 *μ*g/L, *P* = 0.030). Reduction in the average level of FXII in TIC patients than in control was also noted (25.00 ± 12.72 versus 34.71 ± 9.34 *μ*g/L, *P* = 0.049; Figure 2(b)).

### 3.4. Concentrations of FX and FXII Correlate with Clinical Data

As summarized in Table 4, we next analyzed the correlation between FX, FXII, and several key clinical and laboratory data including age, SI, ISS, time to admission, hemoglobin level, platelet count, INR, partial thromboplastin time (PT), activated partial thromboplastin time (APTT), and albumin, all of which can reflect the clinical conditions of patients. As a result, the FX level was shown to have a moderate to strong negative correlation with SI, ISS, APTT, INR, PT, and albumin and a moderate positive correlation with hemoglobin. The FXII level was shown to have a moderate negative correlation with age, INR, PT, and APTT.

### 3.5. MiR-24 Expression Negatively Correlates with the Concentration of FX

**Figure 3:**
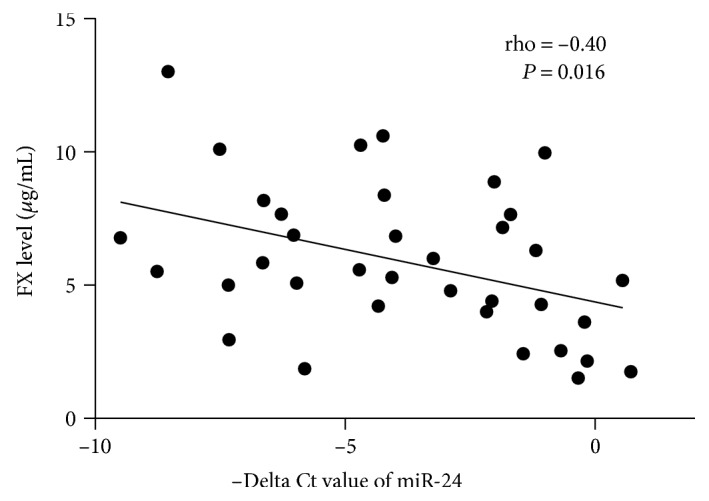
Correlation analysis for miR-24 expression and the level of FX (*n* = 36).

Former studies indicated that miR-24 could reduce the FX and FXII mRNA levels by downregulating HNF-4*α* in liver cancer cell line HepG [22]. Hence, we wanted to determine whether the miR-24 level correlated with FX or FXII levels in trauma patients on their ED arrivals. Pearson's correlation test showed the expression levels of miR-24 were inversely correlated with levels of FX (rho = −0.40, *P* = 0.016; Figure 3) but not with FXII (rho = −0.24, *P* = 0.164).

### 3.6. MiR-24 Can Inhibit FX Synthesis in Human Hepatocyte Cell Line

To investigate the effect of miR-24 on FX of the supernatant of L02 cells, we altered the miR-24 level in the cells. After transfection of miR-24 mimics or negative control, ELISA analysis showed that compared to negative control, the level of FX was reduced by transfection of miR-24 mimics, from (55.38 ± 5.78) ng/mL to (43.30 ± 8.49) ng/mL (*P* = 0.030).

## 4. Discussion

Despite dozens of years of intense research in this field, the management of TIC remains largely supportive without the therapeutic breakthroughs. The present study showed that the plasma miR-24 level was significantly elevated in major trauma patients, especially in TIC patients. It reduced to under average value in 3 days after homeostasis. The level of miR-24 was inversely correlated with that of FX. In vitro study showed that miR-24 transduction significantly reduced the FX level in the supernatant of L02 cells. They altogether indicated that overexpression of miR-24 was involved in the hypocoagulation state by inhibiting the production of FX after severe trauma. This is the first study which tries to explore the relationship between miRNA and coagulation factors in the trauma setting.

miRNAs can target hundreds to thousands of proteins and significant differences in the expression levels are altered in almost every type of disease [28]. Downregulation of miR-24 has been detected in the procoagulant states including myocardial infarction [29], acute cerebral infarction [27], osteomyelitis [30], and rats' acute respiratory distress syndrome [31]. These findings suggested that miR-24 might have an anticoagulant function. A study by Uhlich et al. revealed that the miR-24 level was increased among the 69 differentially expressed miRNAs in the severely injured [21]. In the present study, we confirmed that the miR-24 was overexpressed in major trauma patients. We further displayed that the difference in the miR-24 increase was associated with TIC, which implied that miR-24 might be a useful diagnostic marker for TIC.

FX is synthesized in the liver and requires vitamin K. It plays a pivotal role at the convergence of the intrinsic and extrinsic pathways in the coagulation cascade [32]. Congenital FX deficiency can result in severe bleeding disorders [33]. A recently published cohort study proved that the activity of FX could predict the 28-day mortality after controlling for confounding factors following trauma [34]. Our measurement of FX in the injured by ELISA, which can determine the exact quantification of FX and activated FX combined, was not reported before. We showed that FX concentration was clearly reduced in TIC patients, which was consistent with previous reports showing the decrease of the activity of multiple coagulation factors after major trauma [11, 13]. The FX level was negatively correlated with ISS, SI, and positively with the hemoglobin level, which implied that the FX level might aggravate with the severity of the disease [35, 36]. Our findings added to the current evidence of coagulation factor deficiency after major trauma and provided another proof for the rationale of the use of high ratio of FFP and PCC for the supplement of the depleted coagulation factors.

The liver is the primary source of a number of circulating coagulation factors. Dramatic changes in the homeostasis of the body can gravely impair its synthetic function. For example, fibrinogen production reduces to half after 2 hours of hypermetabolic state [37]. The disturbed coagulation system can be detected early as a result of reduced clotting factor production as evidenced by severe bleeding in acute hepatic failure [38]. miRNAs play a critical role in the fine-tuning of fundamental biological liver processes including glucose, lipid, iron, and drug metabolism [39]. However, studies regarding miRNAs and liver in the coagulation factor production are scanty [22, 40]. In our study, plasma miR-24 expression was negatively correlated with the FX level in trauma patients, and pretreatment with miR-24 significantly suppressed the level of FX in the supernatant of normal liver cell line L02, which agreed with the result of Salloum-Asfar et al. showing that miR-24 could reduce the FX mRNA level in HepG cell line [22]. Considering the synthesis of coagulation factors by the hepatocytes is an ongoing state which can be affected by miRNAs; our results in combination with others indicated that the elevation of miR-24 in the severely injured may have a role in the development of TIC by inhibiting the synthesis of FX. Therefore, miRNAs can be manipulated as therapeutic agents and miR-24 inhibitor may be developed into a novel therapeutic treatment for patients with TIC.

This study has several limitations. The patients were inconsecutive and the sample size was small; therefore, some of the values only reached borderline significance. The average hours from the scene were 8.1 hours, which is a lot longer than those of similar studies outside China. The main reason was that our hospital is a referral one located in the center of a large city and many patients were transferred from remote parts both inside and outside our province. The relatively long time from the scene to the hospital affected the effect that reduction in synthesis may have in the hypocoagulation state after severe trauma. The exact data on blood transfusion before ED visit was not available, which may be the main reason that the transfusion of PRBC and FFB difference between non-TIC and TIC patients did not reach significance. We did not collect data on lactate and BD values because blood gas analysis is not a routine test unless the patient has decreased oxygen saturation in our ED. Lastly, correlation does not mean causality and further basic studies are warranted to confirm if the reduction in synthesis of coagulation factors can be the cause of TIC.

In summary, we demonstrated that the miR-24 level is upregulated in trauma patients, especially in patients who developed TIC. The elevated miR-24 level is negatively correlated with the decreased level of FX. MiR-24 mimics can significantly reduce FX secretion in the cultured hepatocytes. These findings not only provide new insights from a different point of view into coagulopathy during major trauma but also suggest potential new diagnostic and treatment measures for TIC patients.

## Figures and Tables

**Table 1:** Sequences of RT primers and PCR primers.

miRNA	RT primer		PCR primer
has-miR-24-3p	^∗∗∗^CTGTTC	Forward	CCGTGGCTCAGTTCAGCAG
		Reverse	CAGTGCAGGGTCCGAGGTAT
cel-miR-39	^∗∗∗^CAAGCT	Forward	CGCTCACCGGGTGTAAATCAG
		Reverse	CAGTGCAGGGTCCGAGGTAT

^∗∗∗^Mean sequence of ‘GTCGTATCCAGTGCAGGGTCCGAGGTATTCGCACTGGATACGAC'. RT: reverse transcription.

**Table 2:** Demographics.

Variable	Healthy controls (*n* = 15)	Non-TIC (*n* = 28)	TIC (*n* = 8)	*P* value
Age (y)	42.27 ± 14.59	47.96 ± 12.68	46.50 ± 15.21	0.43
Male, *n* (%)	11 (73.33%)	20 (71.43%)	6 (75%)	0.98
Blunt, *n* (%)	N/A	23 (82.14%)	6 (75%)	0.64
Hours from scene	N/A	8.2 ± 3.1	7.6 ± 4.0	0.80
Injury Severity Score	N/A	25 (16–54)	34 (26–45)	**0.03**
Shock index	N/A	0.74 (0.53–2.00)	0.98 (0.60–1.52)	**0.04**
INR	N/A	1.13 (0.93–1.43)	1.70 (1.56–2.11)	**<0.01**
Transfused, *n* (%)	N/A	17 (60.71%)	8 (100%)	**0.03**
Packed red blood cells (U)	N/A	3.5 (0–20)	9 (0–19)	0.13
Fresh frozen plasma (mL)	N/A	0 (0–1150)	425 (0–2000)	0.14
Platelets (U)	N/A	0 (0–3)	1 (1–3)	**<0.01**
In-hospital mortality, *n* (%)	N/A	2 (7.14%)	2 (33.33%)	0.21
LOS in the hospital (d)	N/A	19.93 ± 14.02	23.63 ± 15.84	0.53
LOS in the ICU (d)	N/A	0 (0–11)	0 (0–9)	0.61

INR: international normalized ratio; LOS: length of stay; *p* < 0.05: values in boldface.

**Table 3:** Multivariate analysis for the association of TIC with ISS, SI, and miR-24 expression level.

Variable	Odds ratio (95%CI)	*P* value
Injury Severity Score	1.25 (1.01–1.54)	**0.04**
Shock index	1.20 (0.80–1.80)	0.37
miR-24	2.86 (1.24–6.60)	**0.01**

*p* < 0.05: values in boldface.

**Table 4:** Correlations of coagulation factor levels with patients' clinical and laboratory data.

	Age	SI	ISS	Time to admission	Hemoglobin	Platelet count	INR	PT	APTT	Albumin
FX	0.26 (0.12)	**−0.39 (0.02)**	**−0.56 (<0.01)**	0.22 (0.20)	**0.44 (<0.01)**	0.21 (0.21)	**−0.59 (<0.01)**	**−0.59 (<0.01)**	**−0.36 (0.03)**	**−0.54 (<0.01)**
FXII	0.24 (0.16)	−0.21 (0.21)	−0.10 (0.58)	0.09 (0.61)	0.27 (0.11)	0.25 (0.15)	**−0.37 (0.03)**	**−0.37 (0.03)**	**−0.35 (0.03)**	−0.20 (0.25)

Table shows Pearson's rank correlation coefficient rho with *P* value (in the bracket). SI: shock index; ISS: Injury Severity Score; INR: international normalized ratio; PT: partial thromboplastin time; APTT: activated partial thromboplastin time.
